# Transdermal Testosterone in Female Hypoactive Sexual Desire Disorder: A Rapid Qualitative Systematic Review Using Grading of Recommendations Assessment, Development and Evaluation

**DOI:** 10.7759/cureus.2401

**Published:** 2018-03-31

**Authors:** Kavitha Ganesan, Yacob Habboush, Senan Sultan

**Affiliations:** 1 Internal Medicine, Orange Park Medical Center; 2 Endocrinology, Orange Park Medical Center

**Keywords:** transdermal, testosterone, female, grade, hypoactive sexual desire, estrogen, menopause, libido

## Abstract

Female hypoactive sexual desire disorder (HSDD) is a multifactorial sexual dysfunction disorder characterized by a decrease in sexual desire and personal distress. HSDD occurs in naturally occurring postmenopausal women or secondary to oophorectomy. Multiple studies have assessed the use of transdermal testosterone (TDT) as a management option for patients with HSDD. Our aim is to assess published studies using the Grading of Recommendations Assessment, Development and Evaluation (GRADE) framework for the quality of evidence regarding testosterone use as a short- and long-term therapy for HSDD. We implemented this qualitative systematic review following the Preferred Reporting Items for Systematic Reviews and Meta-Analyses (PRISMA) checklist. We set a GRADE score of 4 (high evidence) as a cutoff point for the quality measure of published studies assessing the use of TDT in HSDD. The outcomes of interest were the efficacy of TDT on the total number of satisfying sexual activity, number of orgasms, sexual desire and distress level in patients with HSDD. These outcomes were evaluated through Sexual Activity Log (SAL), Profile of Female Sexual Function (PFSF), and Personal Distress Scale (PDS) evaluation tools. Five randomized controlled trials were identified to meet the inclusion criteria. The selected studies were of high evidence based on the GRADE score as two of the studies scored 4 points, the other two studies scored 5 points and one study scored 6 points. All of the high quality selected studies had similar outcomes suggesting high effectiveness for the use of 300 µg/d TDT with or without estrogen for the management of HSDD with minimal side effects. One study showed a trend for higher risk of breast cancer in long-term use (0.37%). The use of 300 µg/d of TDT in surgical and natural menopause is an effective plan to manage HSDD in the short- and long-term. Although side effects are minimal, further prospective research is needed to assess the more severe side effects such as breast cancer in the long-term use of TDT.

## Introduction and background

Female hypoactive sexual desire disorder (HSDD) is a multifactorial sexual dysfunction disorder characterized by a decrease in sexual desire that affects the overall quality of life of the patient and leads to personal distress [1-5]. It is hypothesized that HSDD develops as a result of diminished circulating androgens such as testosterone due to functional decline in production due to either menopause or due to the surgical removal of the ovaries through an oophorectomy [6,7]. Currently, HSDD due to menopause or oophorectomy is managed with estrogen which tends to increase the levels of sex hormone binding globulin (SHBG) which binds to multiple different sex hormones including testosterone. Hence, increased SHBG will lead to a decrease in the level of testosterone available in bloodstreams [6,7].

Multiple studies have found that libido can be improved in patients with HSDD through administering estrogen and testosterone as a combination or even testosterone alone [1-5]. It has also been suggested that transdermal testosterone patches (TDT), as a method of administration, might be more effective than oral route as it allows testosterone to bypass the first pass metabolism and provide a consistent level of hormone in the circulation [8].

The aim of the present study is to assess the efficacy of previous studies evaluating the benefits of TDT as a management plan for HSDD based on a qualitative systematic review. We used Grading of Recommendations Assessment, Development and Evaluation (GRADE) as an evaluation tool to assess the quality of previously published articles. We included only randomized clinical trials. GRADE is an emerging consensus on rating quality of evidence and strength of recommendations. By assessing methodological flaws in a study based on scoring for each category, GRADE also allows us to measure quality by calculating a quantitative score. Table 1 provides the GRADE scores and their interpretations [9].

**Table 1:** GRADE score-quality interpretation. GRADE: Grading of Recommendations Assessment, Development and Evaluation

GRADE Score	Quality	Interpretation
≤1	Very low	Any estimate of effect is highly uncertain
2	Low	Further research is very likely to have an important impact on our confidence in the estimate of effect and is likely to change the estimate
3	Moderate	Further research is likely to have an important impact on our confidence in the estimate of effect and may change the estimate
≥ 4	High	Further research is very unlikely to change our confidence in the estimate of effect

## Review

Methods

Study Design

We implemented this rapid qualitative systematic review following the Preferred Reporting Items for Systematic Reviews and Meta-Analyses (PRISMA) checklist. Two reviewers independently searched by consensus. We systematically searched PubMed, Medline, Ovid, EBSC0 and Clinical Key. We used a controlled vocabulary, including ‘testosterone’, ‘women’, and ‘transdermal’, to which we applied relevant subheading ‘hypogonadism’, ‘menopause’, and ‘hypoactive sexual desire disorder’. We limited our search by using the following filters: Human subjects, English language, and since the year 2000. From the title of the studies, we eliminated the irrelevant ones. We also used reference of some of the studies to identify further resources. The present study is based on a qualitative approach to assess the efficacy of using testosterone TDT to manage HSDD. Therefore, the study design did not include the calculation of the quantitative measures.

Study Selection

We included any published article assessing HSDD in women of 18 years of age or older. We also set a GRADE score of 4 or higher as a cutoff point. Selected studies had to also have used three assessment tools to evaluate the intervention’s efficacy and safety by using the sexual activity log (SAL), profile of female sexual function (PFSF), and personal distress scale (PDS). Studies assessing short- and long-term effects of testosterone were included. We excluded any study that included men, participants younger than 18 years of age, and those studies that did not score 4 or higher on the GRADE framework.

Outcomes

The outcomes of interest were the efficacy of transdermal testosterone patch on the total number of satisfying sexual activity, number of orgasms, sexual desire and distress level in patients with HSDD. The mentioned outcomes are evaluated through SAL, PFSF, and PDS evaluation tools.

Results

Search Results and Study Characteristics

**Figure 1:**
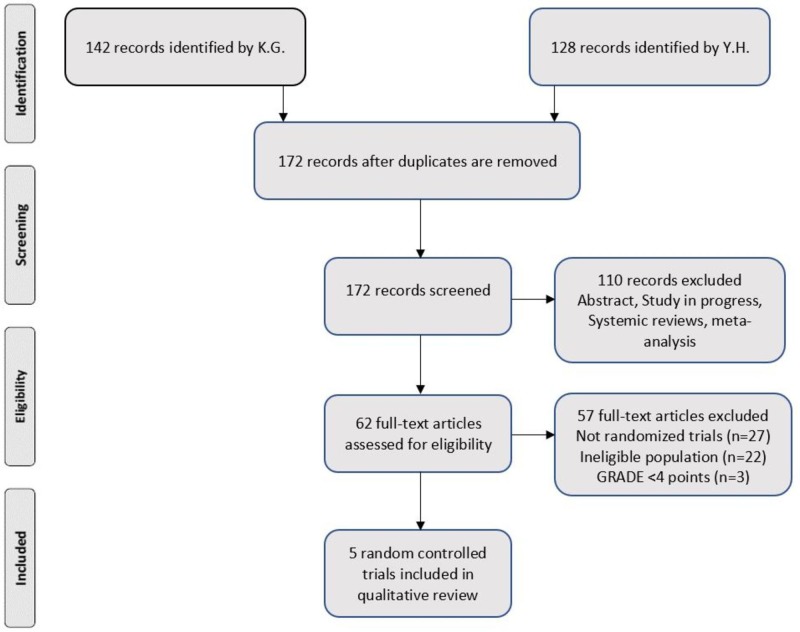
PRISMA flow diagram. PRISMA: Preferred Reporting Items for Systematic Reviews and Meta-Analyses; K.G.: Kavitha Ganesan; Y.H.: Yacob Habboush; GRADE: Grading of Recommendations Assessment, Development and Evaluation.

Both authors Kavitha Ganesan (KG) and Yacob Habboush (YH) cumulatively identified 270 studies between 2008 and 2018. Of these, 98 were duplicates. Hence, 172 studies were screened. One hundred ten studies were excluded as they were irrelevant. Forty-two articles were assessed for eligibility. Thereafter, five randomized controlled trials were identified to be included in the qualitative synthesis. Figure 1 shows the PRISMA flow diagram. Table 2 provides the characteristics of the selected studies.

**Table 2:** Study characteristics of reviewed random controlled trials. RCT: Randomized controlled trial; TDT: Transdermal testosterone; US: United States; UK: United Kingdom

Author/year	Study design	Location	# Cases/ control	Age means (case(s)/control)	Surgical/ Natural menopause	TDT dose (µg/d)	Oral estrogen use	Intervention period (weeks)
Braunstein G. et al. 2005 [1]	RCT	US	328, 119	50.4, 49.6, 49.0, 48.5	Surgical	150, 300, 450	Yes	24
Davis S. et al. 2008 [2]	RCT	US, Canada, Australia, Sweden, UK	537, 277	54.1, 54.3, 54.4	Natural	150, 300	No	52
Panay N. et al. 2010 [3]	RCT	UK, Australia, Germany, Canada	130, 142	56.2, 57.0	Natural	300	No	24
Shifren J. et al. 2006 [4]	RCT	US, Canada, Australia	276, 273	53.9, 54.0	Natural	300	Yes	24
Simon J. et al. 2005 [5]	RCT	US, Canada, Australia	283, 279	49.2, 48.9	Surgical	300	Yes	24

Outcomes

Braunstein et al. [1] evaluated the use of different doses for the TDT in surgically menopausal women. Once compared to control, the 150 µg/d showed no evidence of treatment effect, while 300 µg/d had a statistically significant increase of 67% in sexual desire and frequency of satisfying sexual activity at 24 weeks with p-values of 0.05 and 0.049, respectively (Table 3). However, the trend did not increase significantly with the 400 µg/d TDT. The study concluded that the use of 300 µg/d is the most effective dose of TDT to manage HSDD. This study scored 4 points (high evidence) on the GRADE framework [1].

**Table 3:** Correlations between change from baseline in efficacy of 300 µg/d transdermal testosterone. PFSF: Profile of female sexual function; PDS: Personal distress scale; SAL: Sexual activity log; CI: Confidence interval

Study	Evaluation tools	Mean of change from baseline after 24 weeks [95% CI]	p-value
		Dose: 300 µg/d	
Braunstein G. et al. [1]	PFSF scores - Sexual desire	5.3 [no CI]	0.05
	PDS score	-	0.13
	SAL score - No. of satisfying episodes	8.06 [no CI]	<0.05
Davis S. et al. [2]	PFSF scores - Sexual desire	7 [no CI]	<0.001
	PDS score	-11 [no CI]	<0.001
	SAL score - No. of satisfying episodes	2.1 [no CI]	<0.001
Panay N. et al. [3]	PFSF scores - Sexual desire	7.5 [no CI]	<0.005
	PDS score	-11.52 [-14.58 to -8.46]	0.0024
	SAL score - No. of satisfying episodes	1.16 [0.82-1.5]	0.0089
Shifren J. et al. [4]	PFSF scores - Sexual desire	5.79 [2.82-8.76]	0.0001
	PDS score	-9.04 [-13.49 to -4.58]	0.0001
	SAL score - No. of satisfying episodes	1.38 [0.72-2.03]	<0.0001
Simon J. et al. [5]	PFSF scores - Sexual desire	5.12 [2.20-8.04]	0.0006
	PDS score	-7.70 [-12.14 to -3.26]	0.0006
	SAL score - No. of satisfying episodes	1.11 [0.5-1.73]	0.0003

Davis et al. [2] stated in their study of randomized double-blinded control study that TDT with 300 µg/d resulted in modest improvement in sexual dysfunction and decrease in personal distress (p < 0.001 in 300 mcg of TDT arm and p = 0.04 in 150 mcg of TDT arm) in post-menopausal women who were not receiving estrogen therapy. Incidence of side effects was similar to previous studies except for the higher incidence (30%) of hair growth in TDT arm versus 23.1% in the placebo arm. They reported two patients (0.37%) in TDT arm were diagnosed with breast cancer versus none of them reported in placebo arm over a 52-week period. Davis et al. had the largest number of patients (814 women) in their randomized controlled trial in postmenopausal women. Davis et al. assessed the efficacy of two different doses of TDT in sexually satisfying episodes and concluded that estrogen or combined estrogen and progestin are not required for testosterone to be effective in the treatment of HSDD. This study scored 5 points (high evidence) on the GRADE framework [2].

Panay et al. [3] concluded from their randomized controlled trial that 300 µg/d TDT is effective in terms of improvement of sexual function and treatment of hypoactive sexual desire disorder in naturally menopausal woman irrespective of the use of concurrent hormonal therapy such as estrogen (p = 0.0007). There was also a significant reduction in distress (p = 0.0024) versus placebo at six months. All other endpoints were significantly reduced. These findings represent the real-life situation, as the majority of people in the general population were not taking hormonal therapy for their libido, which was similar to the trials conducted before. The maximum treatment effect was present around 13 to 16 weeks of the clinical trial. Therefore, patients should continue TDT for at least three months before assessing any changes in sexual function. TDT was well tolerated in both groups with no serious side effects during the trial period (24 weeks). Patients with HSDD could have potential improvement in the quality of life while on TDT. This study scored 4 points (high evidence) on the GRADE framework [3].

Shifren et al. [4] mentioned that treatment with TDT of 300 µg/d increased the number of satisfying sexual activity (p < 0.0001), sexual desire (p = 0.0001), and decrease in personal distress (p = 0.0001) in naturally menopausal women with HSDD. They also mentioned that a trial of transdermal testosterone should be implemented at least for a few months before assessing the change in sexual desire in naturally menopause woman, because the change in sexual desire is measured at four to 16 weeks. Also, there was no change in SHBG level while the patients were receiving treatment with transdermal testosterone. On the other hand, the incidence of side effects related to androgen was higher with transdermal testosterone treatment group; however, the side effects were mild. This study scored 6 points (high evidence) on the GRADE framework [4].

Simon et al. [5] assessed the efficacy of 300 µg/d TDT on the frequency of sexual activity at 24 weeks in surgically menopausal women. The study scored 4 points (high evidence) on the GRADE framework. The study suggested a significant increase in the frequency of sexually satisfying episodes from baseline in patients using TDT when compared to control. The effect was present from week 5 with an increase of an average of 2.1 episode/4 weeks with a p-value of 0.0003. Other measures such as the number of orgasms, total activity, and sexual desire all had a significant increase by week 24. While the personal distress level significantly decreased at week 24 with all p-values < 0.005 (Table 3). The study concluded that TDT improved sexual function and decreased distress [5].

All of the five studies reviewed in the current article had an intervention period of 24 weeks and adjuvant therapy of estrogen, except for one study which included 52 weeks intervention and no use of estrogen. The study showed similar results in the long-term use of TDT and without the use of estrogen. All studies showed mild side effect, except for one where a nonsignificant number of women received the diagnoses of breast cancer in the first four months treatment of the study [1-5]. Table 3 shows the correlation of change between placebo and 300 µg/d with all the relevant p-values. Side effects were mostly mild and limited to application site or androgen effects, Table 4 shows the overall percentage of adverse events in all the studies.

**Table 4:** Summary of adverse events during 24 weeks.

Author	Percentage of any adverse events (%)			
	Placebo	Dose: 150 µg/d	Dose: 300 µg/d	Dose: 450 µg/d
Braunstein G. et al. [1]	72.9	-	79.0	-
Davis S. et al. [2]	87.7	84.3	87.6	-
Panay N. et al. [3]	71.1	-	62.3	-
Shifren J. et al. [4]	71.0	77.0	76.0	75.0
Simon J. et al. [5]	79.6	-	77.7	-

Discussion

This qualitative review was undertaken to evaluate the efficacy of TDT to manage HSDD through the use of short- and long-term testosterone patches with or without estrogen in women with natural or surgical menopause. The decrease in production of adrenal and ovarian androgens which starts around the menopausal age in women may affect women’s health significantly along with a decrease in sexual desire [6, 7, 10, 11]. Testosterone affects the female sexual desire through aromatization to estrogen [6, 12].  In our current review and qualitative systemic analysis, we identified five studies highly ranked in accordance to the GRADE framework for quality. In the selected articles all pertained to using TDT as a treatment method for HSDD. As per Davis et al. [2] and Braunstein et al. [1], the dose of 300 µg/d TDT is the most effective dose in the improvement of HSDD in comparison with TDT 150 µg/d and 450 µg/d. Therefore, increase in dose of TDT from 300 to 450 µg/d had no improvement in frequency and desire of satisfying sexual activity when compared to 300 µg/d dose of TDT. It is apparent that the frequency of total satisfactory sexual events significantly increased in all reviewed trials with the use of TDT 300 µg/d with p-value ranging from 0.0001 to 0.05.

Physiologically, testosterone is beneficial in managing HSDD, regardless of the route of administration [7, 13, 14]. However, with the transdermal administration route the side effects were minimal with the most common side effect, a mild rash. Administering testosterone orally may increase the risk for other adverse effects such as blood clots [15].

The reviewed studies (except for Davis et al.) in this review did not assess the long-term safety of testosterone use in postmenopausal women with the risk of breast cancer, body mass index, and cardiovascular adverse effects. Some studies have suggested that the long-term use of testosterone increases the risk of breast cancer in women [16-20]. On the other hand, some studies have indicated that testosterone has no cardiovascular risk and might enhance cardiometabolic function [13, 21]. Stress management, physical exercise, and counselling of the couples may help women with low libido in the absence of any pharmacologic intervention [22-24]. A detailed history of medication use should be obtained before enrolling women in a clinical trial for HSDD as some medications, such as selective serotonin reuptake inhibitors, may influence the outcome of the study as they are known to cause the decrease in sexual arousal, and desire [25-28].

The major strength of this study and what sets it apart from other systematic reviews assessing the use of TDT for HSDD is that we used GRADE framework [9] to select the reviewed articles with a high evidence cutoff, of 4 points or higher, to ensure high-quality outcomes and conclusions. Using the evidence pyramid, where meta-analysis is at the top and basic case reports at the bottom, is outdated and does not reflect the quality of evidence as not every meta-analysis is a high evidence study. One has to assess the different studies making up the analysis to evaluate its quality. Therefore, we ensured that only high-quality studies are included in the current review. Our review is protocol driven which was done in a systematic manner and involved a detailed review of several databases done individually, by two authors to ensure that there was no selection bias and to choose high-quality articles. As our systematic review included the recent publication form the last 20 years which signifies that our systemic review is complete and updated with high evidence.

Few limitations of the present study are due to the design of the study which is a rapid systemic review with limited literature review as we included only the articles from English literature, and transdermal administration of testosterone. We also included studies involving only the postmenopausal women, but generally, women in premenopausal age group could also suffer from HSDD. We also did not include any other systematic review or meta-analysis.

## Conclusions

Transdermal testosterone increases the frequency of sexual desire and sexually satisfying activity in both naturally and surgically menopausal women with HSDD and decreases personal distress. Adjuvant estrogen might not be needed. The evidence is high for short-term use of TDT but less clear for long-term use. Large clinical trials with a longer treatment duration are needed to confirm the long-term efficacy and safety profile of TDT.
